# Extracellular Vesicles Derived From Platelets, Red Blood Cells, and Monocyte-Like Cells Differ Regarding Their Ability to Induce Factor XII-Dependent Thrombin Generation

**DOI:** 10.3389/fcell.2020.00298

**Published:** 2020-05-05

**Authors:** Carla Tripisciano, René Weiss, Sobha Karuthedom George, Michael B. Fischer, Viktoria Weber

**Affiliations:** ^1^Christian Doppler Laboratory for Innovative Therapy Approaches in Sepsis, Department for Biomedical Research, Danube University Krems, Krems, Austria; ^2^Center for Biomedical Technology, Department for Biomedical Research, Danube University Krems, Krems, Austria; ^3^Center for Experimental Medicine, Department for Biomedical Research, Danube University Krems, Krems, Austria

**Keywords:** extracellular vesicles, phosphatidylserine, thrombin generation, coagulation, platelets, flow cytometry

## Abstract

As transmitters of biological information, extracellular vesicles (EVs) are crucial for the maintenance of physiological homeostasis, but also contribute to pathological conditions, such as thrombotic disorders. The ability of EVs to support thrombin generation has been linked to their exposure of phosphatidylserine, an anionic phospholipid that is normally restricted to the inner leaflet of the plasma membrane but exposed on the outer leaflet during EV biogenesis. Here, we investigated whether EVs of different cellular origin and from different settings, namely platelets and red blood cells from blood bank units and a monocyte-like cell line (THP-1), differ regarding their potential to support factor XII-dependent thrombin generation. EVs were isolated from blood products or THP-1 cell culture supernatants using differential centrifugation and characterized by a combination of flow cytometry, nanoparticle tracking analysis, and Western blotting. Soluble factors co-enriched during the isolation of EVs were depleted from blood-cell derived EV fractions using size exclusion chromatography, while proteins bound to the surface of EVs were degraded by mild protease treatment. We found that platelet-derived and red blood cell-derived EVs supported factor XII-dependent thrombin generation to comparable extents, while monocytic EVs failed to support thrombin generation when added to EV-depleted human plasma. We excluded a major contribution of co-enriched soluble proteins or of proteins bound to the EV surface to the thrombogenicity of blood cell-derived EVs. Our data suggest that the enhanced potential of blood cell-derived EVs to support thrombin generation is rather due to enhanced exposure of phosphatidylserine on the surface of blood cell-derived EVs. Extending these investigations to EVs from other cell types, such as mesenchymal stromal cells, will be crucial for their future therapeutic applications.

## Introduction

Extracellular vesicles are sub-cellular fragments originating from the endosomal system or shed from the plasma membrane of virtually all cell types of the human body, generated under physiological or pathological conditions (Deatherage and Cookson, 2012; Sedgwick and D’Souza-Schorey, 2018). EVs are considered key players in intercellular communication, where they exhibit variable functions depending on their cellular origin, their membrane composition, their surface proteins, and their cargo (Valadi et al., 2007; Jorgensen et al., 2015; Willms et al., 2016; McConnell, 2018).

The generic term “extracellular vesicles” encompasses individual vesicle subpopulations, which display overlapping features despite their heterogeneity (Greening and Simpson, 2018). Small EVs (40–130 nm, “exosomes”) originate from the fusion of endosomal multivesicular bodies with the plasma membrane, whereas the formation of large EVs (100–1000 nm; “microvesicles”) involves actin-myosin-based contraction of the cytoskeleton and a rearrangement of plasma membrane phospholipids (D’Souza-Schorey and Clancy, 2012; Hessvik and Llorente, 2017). This phospholipid rearrangement results in the translocation of negatively charged phosphatidylserine from the inner to the outer membrane leaflet, creating a catalytic surface for the attachment and interaction of coagulation factors on the EV surface. Specifically, the assembly of the tenase (factors VIIIa, IXa) and prothrombinase (factors Va, Xa) complexes of the coagulation cascade is greatly facilitated in the presence of EVs, with Ca^2+^ acting as a bridge between negatively charged gamma-carboxyglutamic acid-rich domains on coagulation factors and phosphatidylserine on the EV surface (Furie and Furie, 1992). Thus, phosphatidylserine supports the propagation of coagulation, while tissue factor (TF) or factor XII (FXII) are required as sparks to initiate the coagulation cascade. The exposure of phosphatidylserine on EVs may further contribute to coagulation by supporting the transformation of TF, the high-affinity receptor for FVII/FVIIa and main physiological initiator of the extrinsic coagulation pathway (Mackman, 2009), from a quiescent form into a biologically active state (Chen and Hogg, 2013; Ansari et al., 2016; Grover and Mackman, 2018).

The pro-coagulant role of EVs is clearly evidenced by the presence of elevated levels of EVs in pathologies associated with an increased risk of thromboembolic events, such as cancer, atherosclerosis, and sepsis (Levi et al., 2013; Matsumoto et al., 2015; Deng et al., 2019; Lacroix et al., 2019). Moreover, individuals with impaired phosphatidylserine exposure and EV release, known as Scott syndrome, suffer from severe bleeding disorders (Zwaal et al., 2004).

We have previously shown that platelet-derived EVs from healthy individuals support the propagation of coagulation only under conditions allowing for FXII-driven contact activation (Tripisciano et al., 2017). In the same study, we observed that EVs derived from lipopolysaccharide-stimulated monocyte-like THP-1 cells were able to initiate coagulation independent of contact activation due to their expression of TF, while EVs from unstimulated monocyte-like cells failed to initiate coagulation even under conditions allowing for contact activation. This different ability of EVs derived from platelets and unstimulated monocyte-like cells to support coagulation under conditions allowing for contact activation led us to hypothesize that EVs from different cell types or from different environments, such as blood *vs*. cell culture settings, differ regarding their pro-coagulant characteristics. In support of this hypothesis, recent evidence from studies on human mesenchymal stromal cell-derived extracellular vesicles suggests that the ability of EVs to trigger coagulation depends on the type and state of their parent cells (Christy et al., 2017; Chance et al., 2019).

In the present study, we therefore assessed the ability of EVs derived from blood products (human medical grade platelet concentrates and red blood cell concentrates) and from THP-1 cell culture supernatants as a model for human monocytes (Bosshart and Heinzelmann, 2016), to support coagulation in the presence or absence of contact activation. We were able to confirm differences in the pro-coagulant activity of EVs from different sources, which appear to be due to differential exposure of phosphatidylserine rather than to the presence of soluble factors or EV surface proteins co-enriched during the isolation of EVs.

## Materials and Methods

### Blood Products

Blood collection was approved by the Ethical Review Board of Danube University Krems, and written informed consent was obtained from all donors. Human whole blood was drawn from healthy volunteers into vacutainer tubes containing sodium citrate (Vacuette, Greiner Bio-One, Kremsmuenster, Austria) or sodium citrate supplemented with 50 μg/mL CTI (Haematologic Technologies, Essex Junction, VT, United States) to inhibit contact activation. Freshly drawn blood was centrifuged at 2,500 × *g* for 10 min to remove blood cells, the resulting plasma was collected, centrifuged at 100,000 × *g* for 60 min, sterile filtered to deplete EVs (Minisart 0.2 μm syringe filter, Sartorius Stedim Biotech, Göttingen, Germany), and stored at −80°C until further use in thrombin generation experiments as described below. The efficiency of EV depletion is shown in Supplementary Figure S1.

Medical grade platelet concentrates as well as red blood cell concentrates from healthy volunteer donors were obtained from the Clinic for Blood Group Serology and Transfusion Medicine, Medical University Vienna, Austria, after approval by the local ethics committee (ECS2177/2015). They were produced in a blood bank setting using a Trima Accel^®^ automated blood collection system (Version 5.0, Terumo BCT, Lakewood, CO, United States). Platelet concentrates were stored in polyolefin bags in SSP^+^ solution (Macopharma, Tourcoing, France) at a ratio of 80% SSP^+^ and 20% plasma and used within 2 days. Red blood cell concentrates were stored in polyvinyl chloride (PVC) bags plasticized with di-2-ethylhexyl phthalate in the presence of citrate phosphate dextrose (CPD) supplemented with a combination of sodium chloride, adenine, glucose and mannitol (D’Amici et al., 2012) and used within 17 days.

### Reagents and Cell Culture Media

Phosphate buffered saline without calcium and magnesium (PBS; Life Technologies, Paisley, United Kingdom) was centrifuged at 100,000 × *g* for 60 min and sterile filtered (0.1 μm; Millex-VV Syringe Filter Unit, Merck KGaA, Darmstadt, Germany). Polyacrylamide gels (4–20%), running buffer, sample buffer, and nitrocellulose membranes for Western blotting were obtained from BioRad (Hercules, CA, United States). RIPA buffer (125 mM Tris pH 7.6, 750 mM NaCl, 5% Igepal CA-630, 5% sodium deoxycholate, 0.5% SDS) was purchased from Cell Biolabs (San Diego, CA, United States). RPMI-1640 medium was supplemented with 20 mM 4-(2-hydroxyethyl)-1-piperazineethanesulfonic acid (HEPES), 100 units/mL penicillin and 100 μg/mL streptomycin (all from Sigma Aldrich, St. Louis, MO, United States). Fetal bovine serum (FBS) and human AB serum (both from Sigma Aldrich), were heat-inactivated at 56°C for 30 min and sterile filtered prior to use. AB serum was additionally centrifuged at 100,000 × *g* for 60 min to deplete EVs. All antibodies and fluorochrome-conjugated antibodies used for flow cytometric characterization of EVs and for Western blotting, their respective clones, and their suppliers are specified in Table 1.

**TABLE 1:** Antibodies and fluorochrome conjugates used for flow cytometry and Western blotting.

**Flow cytometry**					

**Antigen**	**Clone**	**Marker for**	**Fluorochrome**	**Abbreviation**	**Supplier**

CD41	P2	platelets	Phycoerythrin Cyanin 7	PC7	Beckman Coulter
CD235a	HIR2 (GA-R2)	red blood cells	Allophycocyanin Alexa Fluor 750	APC AF750	Beckman Coulter
CD45	J33	leukocytes	Pacific Blue	PB	Beckman Coulter
lactadherin	n.a.	phosphatidylserine	Fluorescein Isothiocyanate	FITC	Haematologic Technologies, Inc.

**Western blotting**					
**Antigen**	**Clone**	**Supplier**			

CD63	T63	Invitrogen			
Alix	OTI1A4	Bio-Rad			
α-Actinin1	Sc-17829	Santa Cruz Biotechnology			
Calnexin	polyclonal	Bio-Rad			
Factor IX	polyclonal	Coachrom			
Factor X	polyclonal	Coachrom			

*n.a., not applicable.*

### Cell Culture

Monocyte-like cells (THP-1; American Type Culture Collection), were grown in humidified atmosphere at 37°C and 5% CO_2_ in supplemented RPMI medium containing 10% EV-depleted FBS. When stable growth was reached, cells were harvested by centrifugation at 450 × *g*, washed, and 1 × 10^6^ cells/mL were seeded into supplemented RPMI medium containing 10% AB serum and grown for 4 h. Thereafter, the cell suspension was centrifuged at 450 × *g* for 5 min to deplete cells, and the remaining supernatant was further centrifuged (1,500 × *g*, 15 min, 4°C) to remove debris. The remaining supernatant was collected and immediately used for EV isolation (section “Isolation of Extracellular Vesicles”).

### Isolation of Extracellular Vesicles

Platelet concentrates used to isolate pEVs contained 2.1 ± 0.4 × 10^6^ platelets/μL, 0.06 ± 0.03 red blood cells/μL, and 0.03 ± 0.02 leukocytes/μL. Red blood cell units serving as starting material for the isolation of rbcEVs contained 6.7 ± 0.8 × 10^6^ red blood cells/μL, while platelet and leukocyte counts remained below the detection limit. Platelet concentrates or red blood cell concentrates (section “Blood Products”) were centrifuged at 2,500 × *g*, for 15 min at room temperature (platelets) or at 4°C (red blood cells) to deplete cells and debris. Depletion of blood cells was confirmed by cell counting (Sysmex KX-21N, Sysmex, Neumuenster, Germany), and supernatants were immediately used for EV isolation. To obtain platelet-derived EVs (pEVs), red blood cell-derived EVs (rbcEVs), and EVs derived from monocyte-like cells (mlEVs), the cell-free supernatants from platelet concentrates, red blood cell concentrates, and THP-1 cell cultures obtained as described above were centrifuged at 20,000 × *g* (30 min, 4°C), using a Sorvall Evolution RC centrifuge, Rotor SS-34 (Thermo Fisher Scientific, Waltham, MA, United States). The remaining supernatants were discarded, and the pellets were washed with PBS, re-centrifuged at 20,000 × *g*, and re-suspended in PBS. The protein content of the EV suspensions was determined using the DC Protein Assay (Bio-Rad). Aliquots standardized to a protein content of 4 mg/mL were stored at −80°C until further use.

### Flow Cytometric Characterization of Extracellular Vesicles

Flow cytometry was used to characterize platelet-derived, red blood cell-derived and monocytic EVs obtained as described in section “Isolation of Extracellular Vesicles.” EV suspensions were diluted in PBS to a protein concentration of 1 μg/mL and stained for 15 min at room temperature in the dark with FITC-conjugated lactadherin as marker of phosphatidylserine, as well as with CD41-PC7 as platelet marker, CD235a-APC as red blood cell marker and CD45-PB as leukocyte marker. All antibody conjugates were centrifuged at 17,000 × *g* for 10 min at room temperature prior to use. Stained samples were diluted 5-fold in PBS and analyzed on a Gallios flow cytometer (Beckman Coulter, Brea, CA, United States) equipped with 405, 488, and 638 nm lasers. Fluorescent-green silica particles (1.0, 0.5, 0.3 μm; excitation/emission 485/510 nm; Kisker Biotech, Steinfurt, Germany) were used for calibration, the triggering signal was set to forward scatter/size, and the EV gate was set at the 1 μm bead cloud. EVs were defined as lactadherin-positive events in the EV gate as previously described (Tripisciano et al., 2017; Weiss et al., 2018). The lower size limit of detection was 250 nm. Data were acquired for 3 min at a flow rate of 10 μL/min and analyzed using the Kaluza Software (Beckman Coulter), *n* = 10. Isotype controls and single stainings of specific monoclonal antibodies are shown in Supplementary Figure S2. As no isotype control is available for lactadherin staining, unfiltered PBS, which contains non-defined particular material, was “stained” with lactadherin-FITC to rule out unspecific binding.

### Nanoparticle Tracking Analysis

Particle size range and concentration were assessed by nanoparticle tracking analysis (NTA; Zeta View, Particle Metrix, Inning, Germany) equipped with a 520 nm laser and a 550 nm filter. To assess particle size, EV suspensions (section “Isolation of Extracellular Vesicles”) were diluted 5,000-fold in PBS for scattering mode analyses. Measurements were performed in triplicates at room temperature at a camera sensitivity of 80%, counting an average of 1,000 tracks with 15 fps. Alternatively, we performed measurements in fluorescence mode after staining with the membrane dye Cell Mask Orange (CMO, excitation/emission 554/567 nm, Invitrogen, Carlsbad, CA, United States). EVs were incubated with CMO (5 μg/mL) for 30 min in the dark (EV-to-CMO ratio of 1:2, 10-fold dilution of EVs in PBS). Stained EVs were further diluted 100-fold in PBS and analyzed at a camera sensitivity of 90%. A correction factor of 1.43 was introduced to compare measurements recorded at different sensitivities (80% for scattering mode, 90% for fluorescence mode). Data were analyzed using the Zeta View version 8.04.02. Eleven different EV batches were tested.

### Characterization of Extracellular Vesicles by Western Blotting

Extracellular vesicles fractions (section “Isolation of Extracellular Vesicles”) containing 10 μg protein were added to cold RIPA buffer to a final volume of 10 μL, and the mixture was incubated 5 min on ice and vortexed at maximum speed. Protein extracts (10 μg protein per lane) were resolved by SDS-PAGE under reducing conditions, with the exception of CD63, which was analyzed under non-reducing conditions. Gels were blotted onto nitrocellulose membranes, and blots were developed using anti-CD63, anti-α-Actinin1, anti-Alix, and anti-Calnexin antibodies, as specified in detail in Table 1. To test for the presence of coagulation factors IX (FIX) and X (FX) in blood product-derived EVs, blots were developed with anti-FIX and anti-FX antibodies. Human FIX and FX (Coachrom, Vienna, Austria) as well as human EV-depleted citrate plasma were used as controls. Membranes were developed using the Western Breeze chemiluminescence kit (Invitrogen) according to the manufacturer’s protocol.

### Size Exclusion Chromatography to Deplete Soluble Proteins

To separate EVs from co-enriched soluble proteins, pEVs as well as rbcEVs were subjected to size exclusion chromatography. EV aliquots (400 μg protein) were diluted to 500 μL in PBS and run through 5 mL size exclusion columns (qEVoriginal, Izon Science, Medford, MA, United States), according to the manufacturer’s instructions. Fractions (0.5 mL each) were collected and characterized regarding their protein content (DC assay, Bio-Rad) and their exposure of phosphatidylserine, CD41, and CD235a (FC). Fractions containing LA^+^ events in FC (i.e., EVs exposing phosphatidylserine) were pooled, the pool was re-centrifuged (20,000 × *g*, 30 min), and the resulting EV pellet was re-suspended in PBS and immediately used for further analysis. Three independent experiments were conducted.

### Protease Treatment of Extracellular Vesicles

To degrade membrane-associated factors potentially contributing to thrombin generation, such as coagulation factors associated with the EV surface, EVs were exposed to mild protease treatment. Aliquots of isolated EVs (100 μg protein/mL) were incubated with increasing concentrations of trypsin (0, 1, 5, 10, 50, 100 μg/mL final concentration; Serva, Heidelberg, Germany) at 37°C for 10 min. The reaction was stopped by the addition of protease inhibitor (cOmplete Mini, Roche, Mannheim, Germany), EVs were pelleted at 20,000 × *g*, washed with PBS, re-centrifuged, re-suspended in PBS, and immediately used for further analysis; *n* = 4.

### Thrombin Generation Assay

The pro-coagulant potential of pEVs, rbcEVs, and mlEVs was assessed by thrombin generation assays (Technoclone, Vienna, Austria) as previously described (Tripisciano et al., 2017). In brief, isolated EVs (25 μg protein/mL) were added to EV-depleted human plasma (section “Blood Products”) anticoagulated with citrate in the presence or absence of CTI. The thrombin-dependent cleavage of a fluorogenic substrate was recorded at 37°C for 60 min at 1 min intervals (Synergy 2 microplate reader, Bio-Tek Instruments Inc., Winooski, VT, United States) at an excitation/emission 360/460 nm, and thrombin concentration was calculated after calibration against a thrombin standard; experiments were repeated from 3 to 4 times. Data were analyzed with the Bio-Tek Gen5 software.

### Statistical Analysis

GraphPad Prism version 7.02 (La Jolla, CA, United States) was used for statistical analysis. Data are presented as mean of three or more independent experiments ± standard deviation (SD). For multiple comparisons of normally distributed data, repeated measures one-way analysis of variance (ANOVA) followed by a Bonferroni correction for multiple comparisons were used. A Kruskal-Wallis test followed by Dunn’s multiple comparisons test was used for not-normally distributed data. Values < 0.05 were considered as statistically significant.

## Results

### Characterization of Extracellular Vesicles Derived From Platelets, Red Blood Cells, and Monocytic THP-1 Cells

**FIGURE 1:**
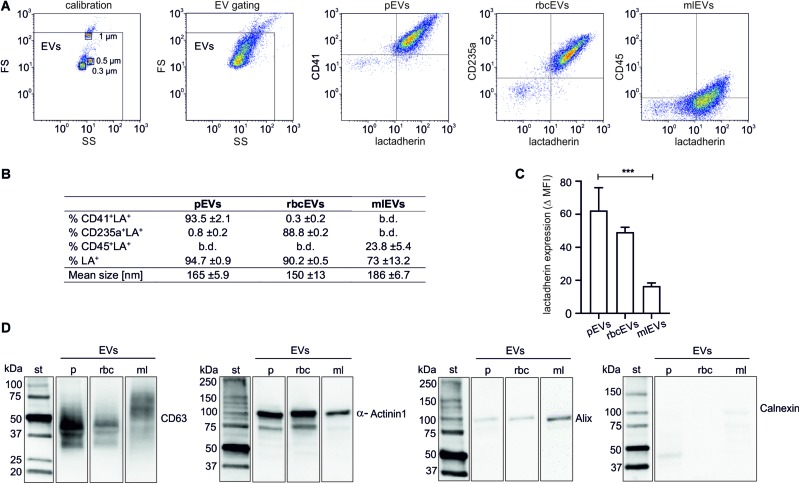
Characterization of extracellular vesicle fractions. EVs were isolated from platelet concentrates (pEVs), red blood cell concentrates (rbcEVs), or from monocytic THP-1 cells (mlEVs). Characterization of isolated EV fractions was performed using flow cytometry, nanoparticle tracking analysis, and Western Blotting, as described in the section “Materials and Methods.” **(A)** Flow cytometric characterization of EVs was performed after calibration with fluorescent silica beads. EVs were defined as phosphatidylserine-exposing (i.e., lactadherin-positive) events in the EV gate, and their cellular origin was assessed using CD41, CD235a, and CD45 as markers for pEVs, rbcEVs, and mlEVs, respectively. **(B)** Exposure of markers of cellular origin (*n* = 10) and mean size of pEVs, rbcEVs, and mlEVs (*n* = 11); b.d., below detection. **(C)** Difference between the specific antibody staining and the respective fluorochrome-labeled reagent control (median fluorescence intensity, MFI) for lactadherin staining. Significance (***p ≤ 0.001) was calculated for pEVs *vs*. mlEVs (*n* = 10); as only two batches of rbcEVs were available, significance cannot be indicated for rbcEVs *vs*. mlEVs. **(D)** Characterization of pEVs, rbcEVs, and mlEVs using Western blotting (10 μg of protein per lane). The blot images are presented as grouped images from different parts of the same membrane or from different membranes. The original blots are available online as Supplementary Datasheet S2 of the Supplementary Material. b.d., below the limit of detection.

Isolated EV fractions were characterized regarding their protein content, particle size and number, the presence of EV protein markers, as well as the exposure of phosphatidylserine and of markers of cellular origin (Figure 1). According to FC, 94.7 ± 0.9% (pEVs), 90.2 ± 0.5% (rbcEVs), and 73 ± 13.2% (mlEVs) of all events in the EV gate exposed phosphatidylserine, as indicated by their positive lactadherin staining. 93.5 ± 2.1 and 88.8 ± 0.2%, of pEVs and rbcEVs, respectively, carried CD41 (platelet marker) or CD235a (red blood cell marker), while only 23.8 ± 5.4% of mlEVs stained positive for CD45 (leukocyte marker; Figures 1A,B). Monocyte-like THP-1 cells were 100% CD45^+^ (Supplementary Figure S3), indicating that CD45 is not fully transferred to mlEVs from their parent cells. Alternatively, antibodies may differ in their ability to detect molecules on cell surfaces vs. EVs due to conformational changes of surface molecules on strongly curved surfaces, leading to apparently lower expression of CD45 on EVs *vs*. cells.

To obtain an estimate of phosphatidylserine exposed on the surface of pEVs, rbcEVs, and mlEVs, we calculated the difference between specific lactadherin signals for each vesicle subtype and fluorochrome-labeled reagent controls (delta median fluorescence intensities; MFI) and found that pEVs (delta MFI 62.4 ± 13.8) and rbcEVs (49.3 ± 2.9) exhibited higher surface expression of phosphatidylserine as compared to mlEVs (16.5 ± 2.0). The difference was statistically significant for pEVs *vs*. mlEVs (both *n* = 10; Figure 1C), while the number of available rbcEV batches (*n* = 2) for this specific experiment did not allow for the calculation of statistical significance.

The mean particle size, according to nanoparticle tracking analysis, was 161 ± 5.9 nm for pEVs, 140 ± 13 nm for rbcEVs and 186 ± 6.7 nm for mlEVs. In Western blotting (Figure 1D), the tetraspanin CD63, which is highly glycosylated and predominantly associated with late endocytic organelles (Kowal et al., 2016; Doyle and Wang, 2019), was present as a band of 30–60 kDa in pEVs and, at lower abundance, in rbcEVs, while it appeared as band of 50–70 kDa in mlEVs, presumably due to differential glycosylation. α-Actinin1, a marker of medium-sized to large EVs (Takov et al., 2019) mediating the attachment of actin filaments to the cell membrane at adherence-type junctions (Otey and Carpen, 2004) was abundantly present in EVs from all three cellular sources. Alix, an accessory protein of the endosomal sorting complex required for transport (ESCRT) involved in the biogenesis and cargo sorting of vesicles at late endosomes (Iavello et al., 2016) appeared as weak band in mlEVs, and was barely detected in pEVs and rbcEVs. We failed to detect Calnexin, a protein localized in the endoplasmic reticulum and excluded from EVs regardless of their origin (Théry et al., 2018) indicating the absence of major cellular contaminants in the isolated EV preparations. Individual lanes represent different parts of the same blot or of different membranes.

### Extracellular Vesicles Derived From Platelets, Red Blood Cells, and Monocyte-Like Cells Differ Regarding Their Potential to Induce Thrombin Generation

To examine the ability of pEVs, rbcEVs, and mlEVs to induce coagulation, we assessed thrombin generation induced by the addition of pEVs, rbcEVs, and mlEVs to EV-depleted human plasma. To suppress the activation of coagulation via the contact activation pathway in our experimental set-up, we added CTI, a serine protease inhibitor that specifically prevents the activation of human coagulation factor XII, as previously described (Tripisciano et al., 2017).

**FIGURE 2:**
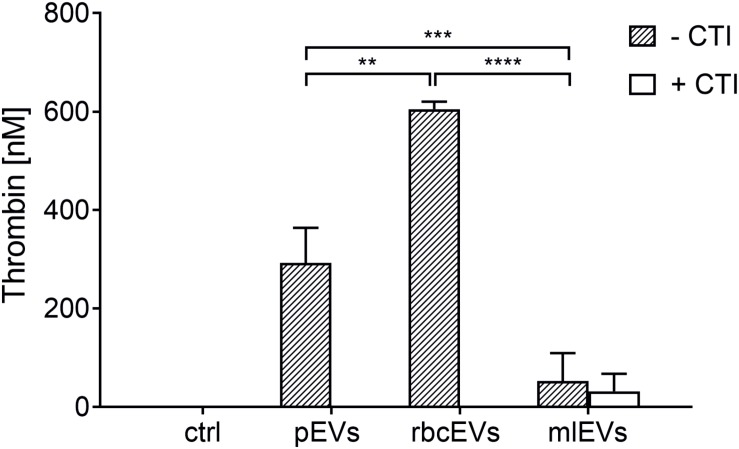
Thrombin generation induced by pEVs, rbcEVs, and mlEVs. EVs were isolated from medical grade platelet concentrates or red blood cell concentrates as well as from monocyte-like cell culture supernatants as described in the section “Materials and Methods.” Aliquots of isolated EVs were normalized regarding their protein content and added to EV-depleted human plasma at a final concentration of 25 μg/mL, in the presence or absence of corn trypsin inhibitor (CTI; 50 μg/mL), an inhibitor of contact activation. Thrombin generation was quantified via the cleavage of a fluorogenic substrate (*n* = 3); ctrl, plasma incubated with buffer only; **p ≤ 0.01; ***p ≤ 0.001; ****p ≤ 0.0001.

Both, pEVs and rbcEVs failed to induce thrombin generation in the presence of CTI, while significant amounts of thrombin were generated under conditions allowing for contact activation, i.e., when CTI was omitted (Figure 2), which is in agreement with previous findings (Rubin et al., 2010; Van Der Meijden et al., 2012). EVs derived from monocytic THP-1 cells, in contrast, did not support coagulation, regardless of the presence or absence of CTI.

### Depletion of Soluble Proteins Fails to Reduce the Thrombogenicity of Platelet-Derived or Red Blood Cell-Derived Extracellular Vesicles

**FIGURE 3:**
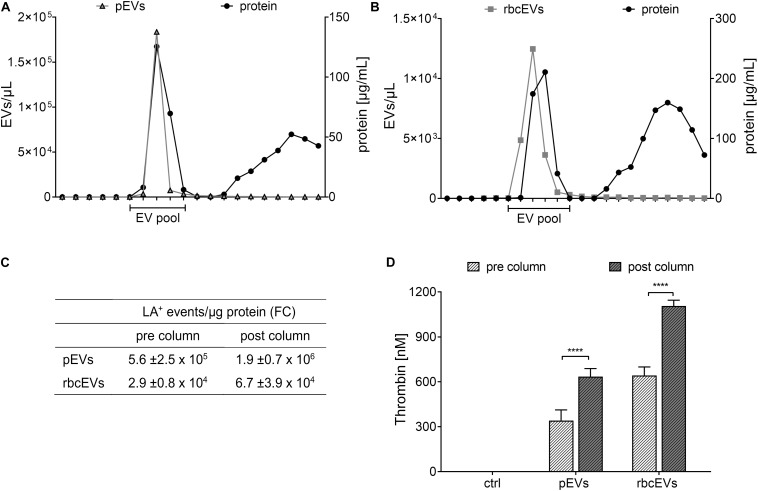
Influence of soluble proteins co-enriched during isolation of extracellular vesicles on thrombin generation. **(A)** EVs isolated from medical grade platelet concentrates (pEVs) and **(B)** from red blood cell concentrates (rbcEVs) were separated from soluble co-enriched proteins by size exclusion chromatography (SEC), and EV-containing fractions were pooled and characterized as described in the section “Materials and Methods.” **(C)** EV counts (assessed by FC) normalized to protein content before and after size exclusion chromatography. **(D)** Thrombin generation induced by pEVs and rbcEVs before and after purification via size exclusion column chromatography (*n* = 3); ****p ≤ 0.0001.

To assess whether soluble proteins co-enriched during the isolation of pEVs and rbcEVs from blood products would account for their thrombogenicity, we further purified pEVs and rbcEVs by size exclusion chromatography. This purification step depleted substantial amounts of soluble proteins, as evidenced by approximately 3-fold higher EV counts per μg protein in the purified EV fractions according to FC (Figure 3). Depletion of soluble proteins did, however, not result in decreased thrombogenicity. On the contrary, SEC-purified pEV and rbcEV fractions induced significantly higher thrombin generation as compared to non-purified fractions, when samples were normalized regarding their protein content (Figure 3D). This reflects the higher concentration of phosphatidylserine-exposing EVs after purification and excludes that soluble proteins co-enriched during EV isolation from blood products are responsible for the thrombogenicity of pEVs and rbcEVs.

### Protease Treatment Degrades Extracellular Vesicle Surface Proteins, but Does Not Result in Reduced Thrombogenicity

**FIGURE 4:**
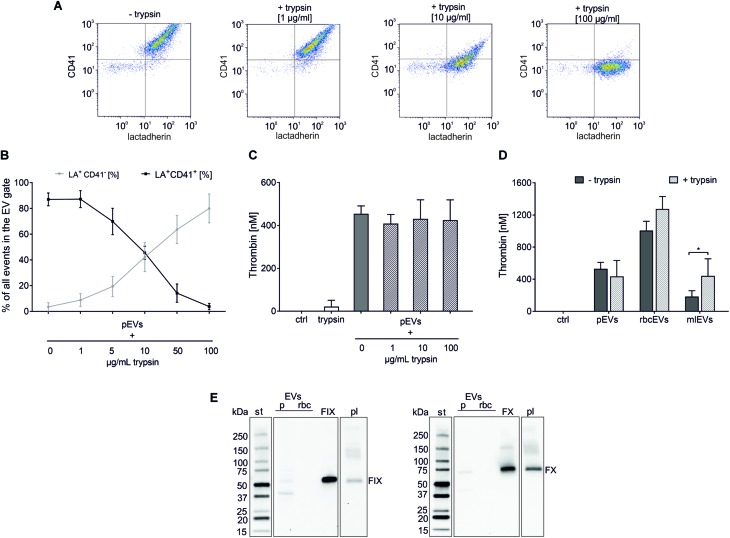
Protease treatment to degrade surface-associated EV proteins. pEVs, rbcEVs, and mlEVs were treated with trypsin to degrade surface-associated proteins as described in the section “Materials and Methods.” **(A)** Flow cytometry density plots of platelet-derived EVs prior to (–trypsin) and after (+trypsin) protease treatment with increasing trypsin final concentrations (1, 10, 100 μg/mL), showing the gradual repositioning of the EV cloud from the CD41^+^ (upper, right) to the CD41^−^ (lower, right) quadrant. **(B)** The effect of trypsin treatment was monitored via the decrease of CD41 on the surface of pEVs, which was dose-dependent (*n* = 4). Exposure of phosphatidylserine remained unaffected by protease treatment, as shown by flow cytometry after staining with fluorochrome-labeled LA. **(C)** Thrombin generation induced by pEVs (25 μg protein/mL) after treatment with 1, 10, and 100 μg/mL trypsin (*n* = 4); trypsin alone was used as control (25 μg/mL). **(D)** Thrombin generation induced by pEVs, rbcEVs, and mlEVs with and without trypsin treatment (100 μg/mL; *n* = 3), *p < 0.05. **(E)** Western blotting confirmed the absence of coagulation factors IX and X in blood-cell derived EV fractions (pEVs and rbcEVs). 10 μg of protein were loaded per well; st, molecular weight standard; FIX, FX, human native factor IX or factor X, 20 ng/lane; pl, EV-depleted citrate plasma diluted 50-fold was used for comparison. The blot images are presented as grouped images from different parts of the same membrane or from different membranes. The original blots are available online as Supplementary Data Sheet S1 of the Supplementary Material.

Having excluded soluble protein contaminants as major source of thrombin generation in pEV and rbcEV fractions, we hypothesized that factors exposed on the surface of EVs, such as coagulation factors, might support thrombin generation induced by EVs derived from platelets and red blood cells. To test this hypothesis, pEVs and rbcEVs were subjected to mild protease treatment, which was monitored via the dose-dependent decrease of CD41 expression on pEVs (Figures 4A,B). Degradation of EV surface proteins did, however, not result in reduced thrombin generation by pEVs and rbcEVs, providing evidence that proteins associated with the surface of pEVs and rbcEVs are not primarily responsible for EV-induced thrombin generation (Figures 4C,D). Of note, protease-treated monocytic EVs even exhibited enhanced thrombogenicity as compared to the untreated control, which will be further addressed in the Discussion.

To explicitly exclude a role of EV-associated coagulation factors in thrombin generation induced by pEVs and rbcEVs, we evaluated the expression of FIX and FX via Western blotting. Using affinity-purified FIX and FX from human plasma as control, we failed to detect FIX and FX in pEV and rbcEV fractions (Figure 4E). Individual lanes represent different parts of the same blot (FIX) or of the same blot at different exposure times (FX).

## Discussion

The ability of EVs to promote and support thrombin generation is widely recognized, underlining their relevance in hemostasis and thrombosis (Rubin et al., 2010; Zwicker et al., 2011; Van Der Meijden et al., 2012; Nomura and Shimizu, 2015).

In a previous study, we found that platelet-derived EVs were able to propagate coagulation initiated by FXII-mediated contact activation in the absence of TF, whereas EVs derived from unstimulated monocyte-like cells failed to support FXII-mediated coagulation (Tripisciano et al., 2017). This prompted us to investigate whether the different ability of platelet-derived and monocyte-like EVs to promote thrombin generation was dependent on differences in their cellular origin and membrane composition or was rather due to factors co-enriched during EV isolation from different settings, such as blood *vs*. cell culture medium. In our present work, we additionally included EVs derived from red blood cells, and found that they supported FXII-dependent thrombin generation to an extent comparable to platelet-derived EVs. This led us to assume that residual plasma-derived soluble proteins or plasma proteins bound to the EV surface - such as coagulation factors - might be mainly responsible for the pro-coagulant activity of blood-cell derived EVs.

We chose to use monocyte-like THP-1 cells rather than primary human monocytes, as the isolation of primary human monocytes would barely have yielded sufficient quantities of monocyte-derived EVs. Moreover, we have recently shown (Fendl et al., 2019) that primary monocytes isolated from whole blood inevitably contain residual platelets (approximate ratio of monocytes:platelets 2:1 using a commercial monocyte isolation kits), and that monocyte preparations therefore contain considerable amounts of platelet-derived EVs, which would have confounded our results.

Co-enrichment of soluble plasma proteins is inevitable when isolating EVs by centrifugation according to their buoyant density, since protein complexes and lipoproteins overlap in size and density with circulating EVs (Konoshenko et al., 2018). Plasma proteins including coagulation factors may also be part of the external cargo or protein corona of EVs derived from blood products. We therefore first depleted soluble co-enriched proteins from isolated EV fractions using size exclusion chromatography and re-evaluated the ability of purified EV fractions to induce thrombin generation. Contrary to our hypothesis, SEC-purified pEVs and rbcEVs generated significantly higher amounts of thrombin as compared to unpurified EV fractions. As we normalized all EV fractions regarding their protein content prior to thrombin generation assays, SEC-purified fractions contained larger numbers of EVs per μg of total protein as compared to unpurified fractions. This provides evidence that thrombin generation depended on the number of EVs per sample and argues against a role of soluble co-enriched factors in the thrombogenicity of blood cell-derived EVs.

We therefore went on to test the role of surface-associated proteins, such as coagulation factors bound to the membrane of EVs. To this end, we exposed pEVs, rbcEVs, and mlEVs to mild trypsin treatment to degrade EV-associated proteins and employed FC to investigate the effect of protease treatment. Incubation with trypsin resulted in a dose-dependent decrease of EV surface protein expression, as monitored for CD41 on platelet-derived EVs. Using this approach, we selected a trypsin concentration of 100 μg/mL for all further experiments to ensure efficient digestion of EV surface proteins as well as maintenance of EV integrity. While trypsin concentrations of up to 1 mg/mL have been recently reported to digest EV surface proteins (Fitzgerald et al., 2018), comparable conditions resulted in significantly decreased EV counts in our experimental setting according to FC, indicating a loss of EV integrity.

When protease-treated EV fractions were subjected to thrombin generation assays, we found that degradation of EV surface proteins did not decrease the potential of pEVs and rbcEVs to induce thrombin generation. Quite on the opposite, a significant augmentation in thrombin generation was observed after protease treatment of monocytic EVs. This could suggest the presence of anti-coagulant factors on monocyte-like cells and, consequently, also on monocytic THP-1 EVs. Monocytes have been reported to express the transmembrane protein thrombomodulin (Satta et al., 1997), which decreases upon stimulation with lipopolysaccharide (Kim et al., 2007). Thrombomodulin, first described on endothelial cells, is an essential cofactor for the activation of the anticoagulant protein C by thrombin. It is thus conceivable that trypsin treatment of mlEVs resulted in the degradation of thrombodulin - or other inhibitors of coagulation - on the mlEV surface, leading to enhanced thrombin generation, but this remains to be further investigated.

In addition to trypsin treatment, which targets the whole spectrum of EV surface proteins, we assessed the presence of specific coagulation factors in EVs derived from blood cells. Factor X induces the cleavage of prothrombin into thrombin, when activated by the TF/FVIIa complex of the extrinsic pathway or by factor IX of the contact pathway. We therefore focused on the presence of FIX and FX in pEV and rbcEV fractions, but were unable to detect either of these factors using Western blotting. Of other potential candidate factors, we excluded FVII, which is exclusively involved in the TF-mediated extrinsic pathway, as well as factors XI and XIIa, which, if present, would have triggered thrombin formation even in plasma anticoagulated with CTI. FXIII, which acts downstream of thrombin and therefore is not relevant in the context of thrombin generation, was also omitted.

Several reports in the literature have associated the procoagulant activity of EVs from red blood cell concentrates to FXI rather than FXII, as FXI is involved in normal hemostasis not only upon activation by factor XIIa and thrombin, but also by self-activation (Gao et al., 2013). The importance of FXI was as well reported by Rubin et al. (2013), who showed that rbcEVs at concentration of ≥ 5 × 10^9^/L induced thrombin generation despite the presence of CTI. Very recently, EVs from red blood cell units have been suggested to initiate the intrinsic pathway by two mechanisms leading to FIX activation, (i) the normal FXIIa-FXI-FIX pathway and (ii) the activation of kallikrein that in turn directly activates FIX (Noubouossie et al., 2020). Notably, the authors of this study described that heating at 60°C or treatment with trypsin abolished the ability of rbcEVs to induce thrombin generation, suggesting that EV-associated proteins were responsible for the activation of FXII and kallikrein.

In our study, protease treatment did not affect the thrombogenicity of rbcEVs or pEVs. It should be noted, however, that the rbcEVs used in the studies by Rubin et al. (2010); Gao et al. (2013), and Noubouossie et al. (2020) were derived from red blood cell units that had been stored for 42–45 days, which is likely associated with oxidative changes and storage lesion (D’Alessandro et al., 2015; Almizraq et al., 2018). This enhanced thrombogenicity upon prolonged storage of red blood cell units may also be the cause of a higher incidence of deep vein thrombosis after transfusion of aged blood units (Koch et al., 2019). One might therefore speculate that prolonged storage of red blood cells induces biochemical modifications on the EV surface, resulting in altered thrombogenicity.

Overall, in the present study, both the results obtained for protease-treated EVs and the lacking expression of coagulation factors contradict a major involvement of EV surface proteins in the induction of thrombin generation. The different ability of pEVs and monocytic EVs to support FXII-mediated thrombin generation might therefore rather be due to differences in their membrane composition. Based on the primary role of phosphatidylserine in supporting coagulation (Lentz, 2003; Ansari et al., 2016; Su et al., 2018), our data provide evidence that EVs from different sources or settings might differ regarding their exposure of phosphatidylserine. This is further supported by the data obtained with flow cytometric characterization of pEVs, rbcEVs, and mlEVs in the present work, showing higher density of phosphatidylserine on pEVs and rbcEVs as compared to mlEVs. Along this line, a recent study found that human pancreas carcinoma and colorectal adenocarcinoma cell lines secrete distinct EV subpopulations of different size, density, surface charge, and phosphatidylserine expression (Matsumura et al., 2019).

## Conclusion

Our study provides evidence that EVs enriched from different settings differ regarding their ability to support thrombin generation. Neither soluble contaminants nor membrane-associated proteins appear to be responsible for the enhanced pro-coagulant potential exhibited by platelet- and red blood cell-derived EVs as compared to cell culture-derived monocytic EVs. Our findings suggest that differences in phosphatidylserine exposure might account for the different ability of EVs from different settings to support thrombin generation. We are currently extending our studies to the pro-coagulant potential of EVs derived from mesenchymal stromal cells, which has important implications for any therapeutic application of these EVs.

## Data Availability Statement

The datasets generated for this study are available on request to the corresponding author.

## Ethics Statement

The studies involving human participants were reviewed and approved by Ethical Review Board of Danube University Krems. The patients or participants provided their written informed consent to participate in this study.

## Author Contributions

CT wrote the manuscript, designed all the experiments and performed data acquisition and analysis on Western blotting, protease digestion, thrombin generation, and size exclusion chromatography. RW performed the characterization of EV fractions by flow cytometry. SK carried out measurements by nanoparticle tracking analysis. VW conceived the study, contributed to data interpretation, and wrote the manuscript. MF provided platelet and red blood cell concentrates and contributed to data interpretation and to critical revision of the manuscript.

## Conflict of Interest

The authors declare that the research was conducted in the absence of any commercial or financial relationships that could be construed as a potential conflict of interest.

## Abbreviations

CTI: corn trypsin inhibitor
EVs: Extracellular vesicles
FC: flow cytometry
FIX: coagulation factor IX
FXII: coagulation factor XII
FX: coagulation factor X
LA: lactadherin
MFI: median fluorescence intensity
mlEVs: monocyte-like cell-derived EVs
NTA: nanoparticle tracking analysis
pEVs: platelet-derived EVs
rbcEVs: red blood cell-derived EVs
TF: tissue factor.
