# Supplementary material for: Extracellular Vesicles Derived From Platelets, Red Blood Cells, and Monocyte-Like Cells Differ Regarding Their Ability to Induce Factor XII-Dependent Thrombin Generation
Source: Front Cell Dev Biol. 2020 May 5;8:298. doi: 10.3389/fcell.2020.00298 (PMC7232549; doi:10.3389/fcell.2020.00298)
Supplement: Supplementary file 1 [file Data_Sheet_1.DOCX]

**Figure 4E – original blots**

**Factor IX**

**
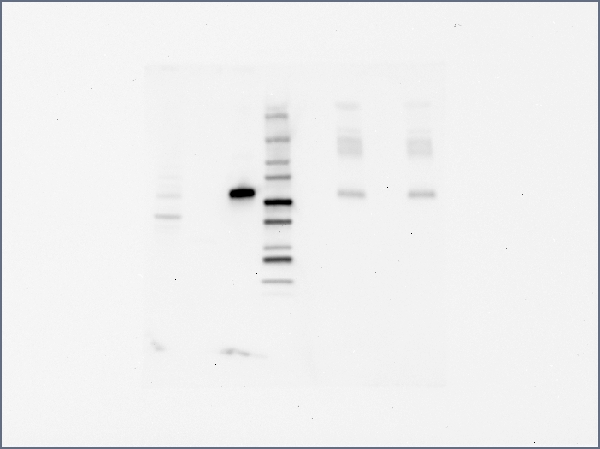
**

FIX

rbc
EVs

pEVs

plasma

**Factor X – samples**

**
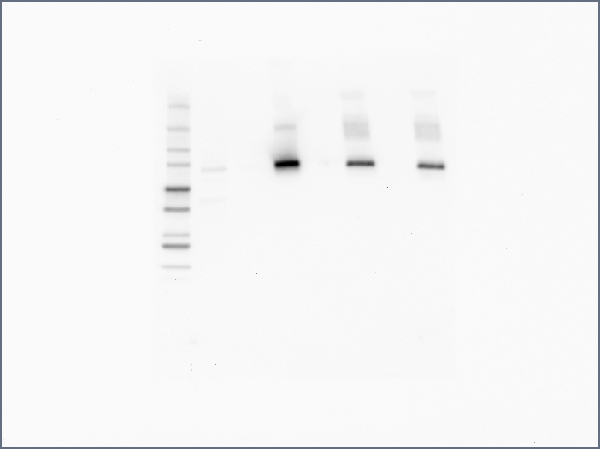
**

plasma

FX

rbc
EVs

pEVs

**Molecular weight standard**

**
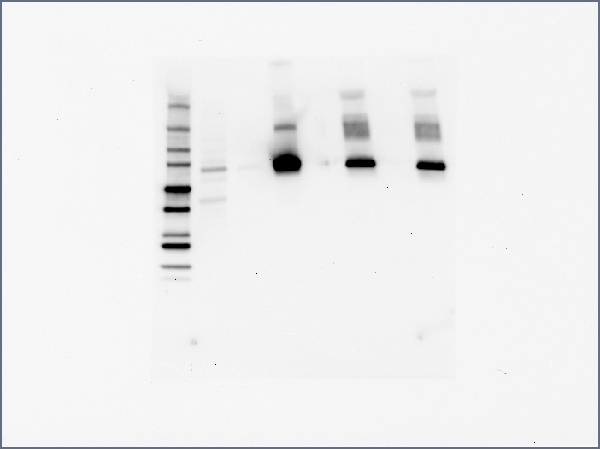
**
