# Supplementary material for: Extracellular Vesicles Derived From Platelets, Red Blood Cells, and Monocyte-Like Cells Differ Regarding Their Ability to Induce Factor XII-Dependent Thrombin Generation
Source: Front Cell Dev Biol. 2020 May 5;8:298. doi: 10.3389/fcell.2020.00298 (PMC7232549; doi:10.3389/fcell.2020.00298)
Supplement: Supplementary file 2 [file Data_Sheet_2.DOCX]

**Figure 1D – original blots**

**CD63 – platelet EVs**

**
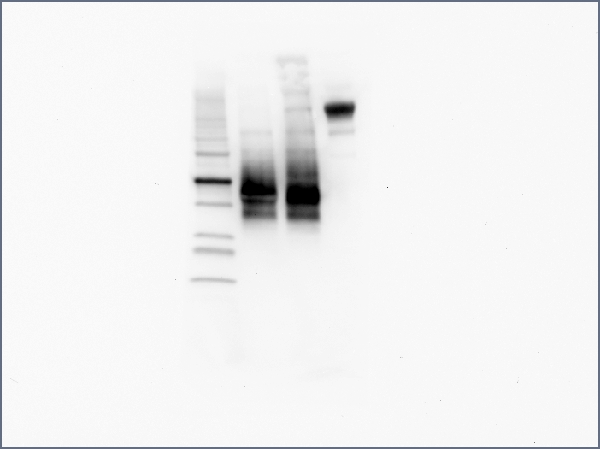
**

pEVs

**CD63 – red blood cell EVs and monocytic EVs**

**
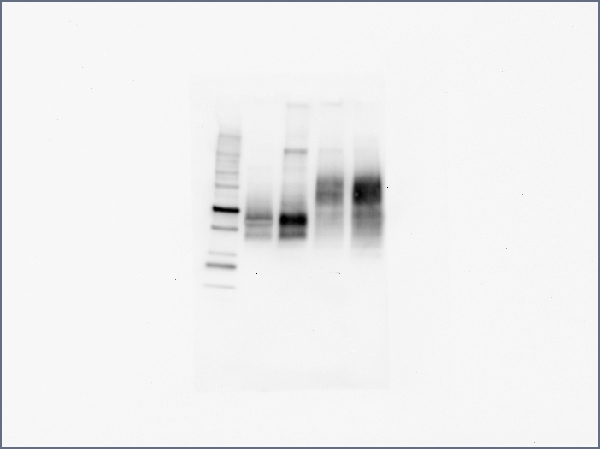
**

mEVs

rbc
EVs

**CD63 - Molecular weight standard**

**
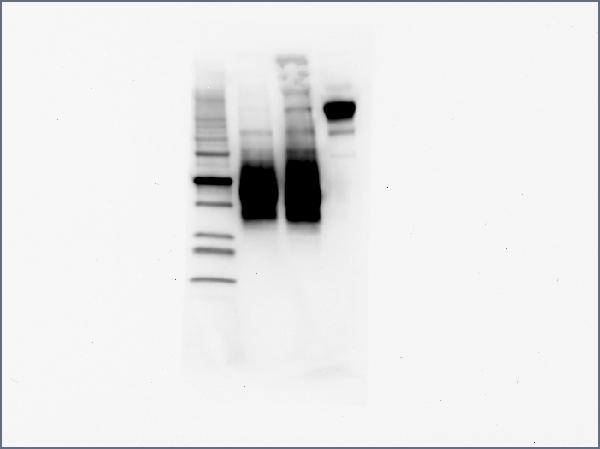
**

**Alix – platelet EVs and monocytic EVs**

**
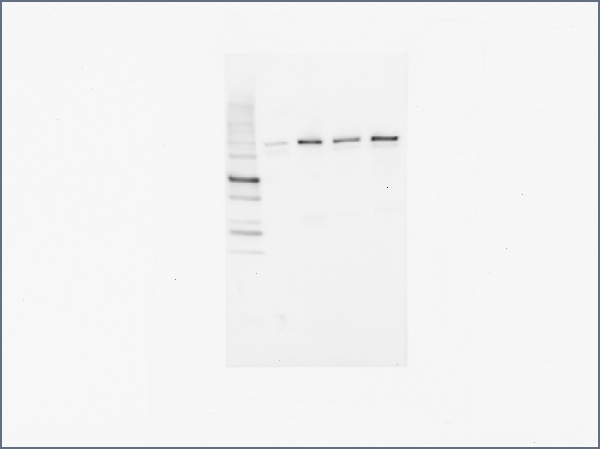
**

mEVs

pEVs

**Alix – red blood cell EVs**

**
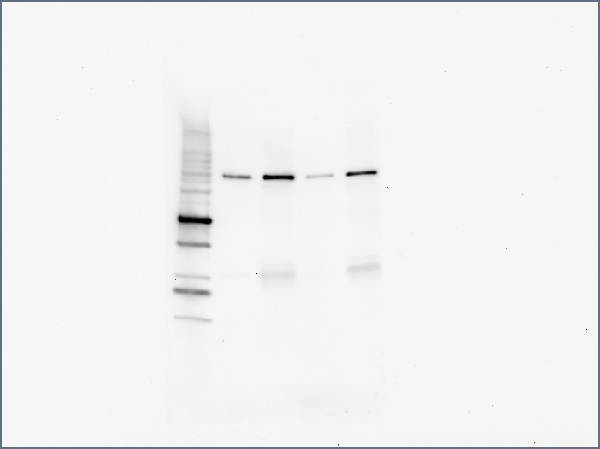
**

rbc
EVs

**Alix - Molecular weight standard**

**
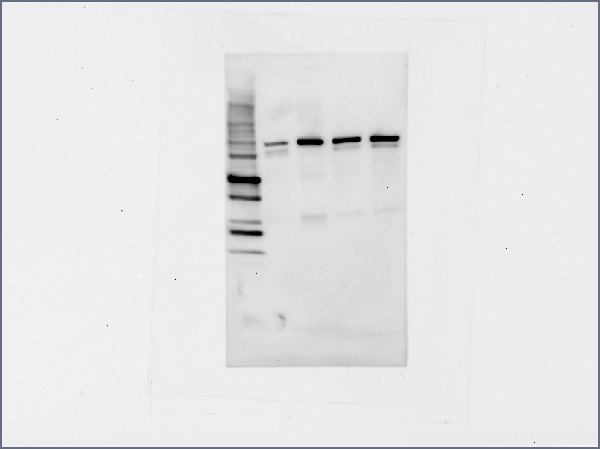
**

**α-Actinin1 – platelet EVs and monocytic EVs**

**
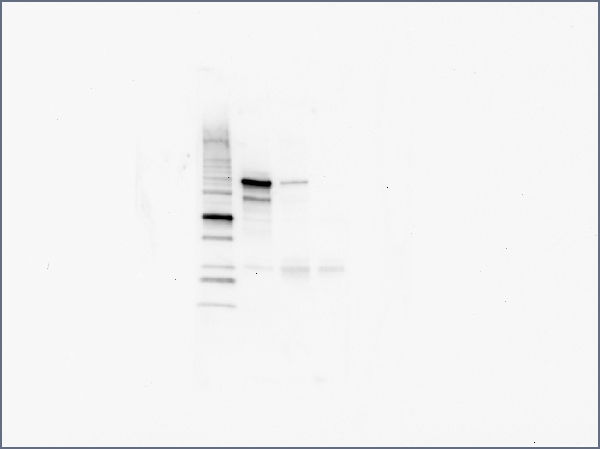
**

pEVs

**α-Actinin1 – red blood cell EVs**

**
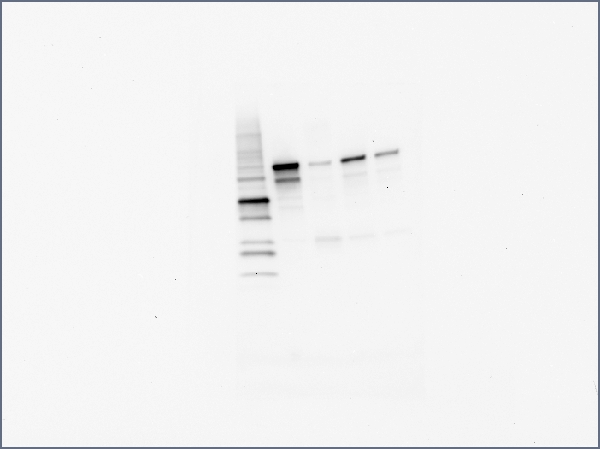
**

**mEVs**

**rbc
EVs**

**α-Actinin1 - Molecular weight standard**

**
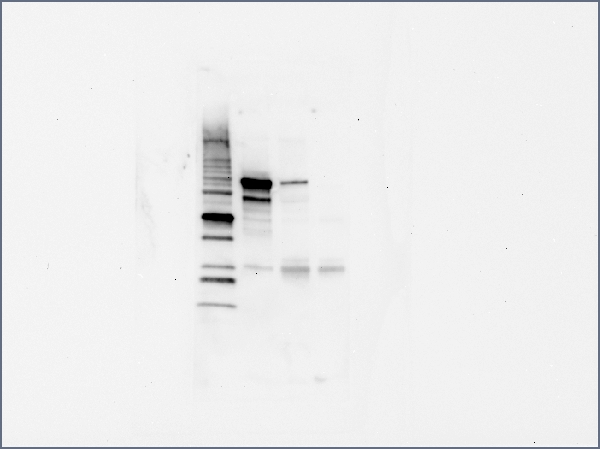
**

**Calnexin – platelet EVs, red blood cell EVs, monocytic EVs**

**
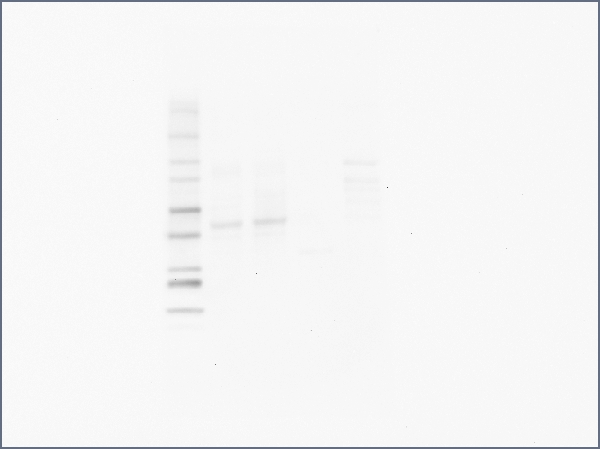
**

mEVs

pEVs

rbc
EVs

**Calnexin- Molecular weight standard**

**
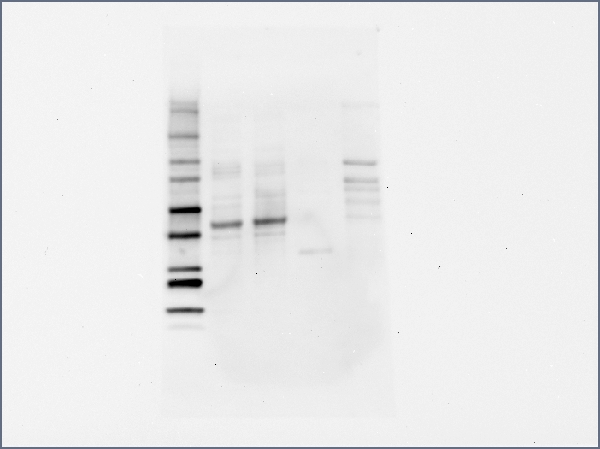
**
