# Supplementary material for: Extracellular Vesicles Derived From Platelets, Red Blood Cells, and Monocyte-Like Cells Differ Regarding Their Ability to Induce Factor XII-Dependent Thrombin Generation
Source: Front Cell Dev Biol. 2020 May 5;8:298. doi: 10.3389/fcell.2020.00298 (PMC7232549; doi:10.3389/fcell.2020.00298)
Supplement: Supplementary file 3 [file Table_1.docx]

Supplementary Material

Supplementary Figures





**Supplementary Figure S1: Flow cytometric characterization of non-EV-depleted and EV-depleted plasma.** (A) Human whole blood was drawn into vacutainer tubes as described in the main manuscript, and plasma was obtained by centrifugation of whole blood at 2,500 g for 10 min, and characterized by flow cytometry as described in the main manuscript. EVs (shown here for platelet-derived EVs) appear in the right upper and lower quadrant of the scatter plot (CD41^+^, LA^+^). (B) Plasma was centrifuged at 100,000 g and sterile filtered to deplete EVs.





**Supplementary Figure S2: Isotype controls and single stainings for the flow cytometric characterization of extracellular vesicles.** To assess the expression of phosphatidylserine on the EV surface, lactadherin was used for labeling. As no isotype control is available for lactadherin, unfiltered PBS, which contains non-defined particular material, was “stained” with lactadherin-FITC to assess unspecific binding. CD41 was used as a platelet marker, CD235a as red blood cell marker, and CD45 as leukocyte marker. The difference between the fluorochrome-labeled reagent control and the specific antibody staining are shown for each marker. Bars indicate positive expression.





**Supplementary Figure S3: Surface expression of CD45 on monocyte-like THP-1 cells.** Staining of cells was performed with CD45-PB as described in the main manuscript. The respective isotype control and single staining are shown.
